# Assessing and improving health in the workplace: an integration of subjective and objective measures with the STress Assessment and Research Toolkit (St.A.R.T.) method

**DOI:** 10.1186/1745-6673-7-18

**Published:** 2012-09-20

**Authors:** Chiara Panari, Dina Guglielmi, Aurora Ricci, Maria Carla Tabanelli, Francesco Saverio Violante

**Affiliations:** 1 Department of Economics, University of Parma, Via Kennedy, Parma, 6 43100, Italy; 2 Department of Science Education, University of Bologna, Via Filippo Re, Bologna, 6 40126, Italy; 3 Department of Internal Medicine, Section of Occupational Medicine Geriatrics and Nephrology Alma Mater Studiorum University of Bologna, Via Massarenti 9 40138, Bologna, BO, Italy

**Keywords:** Observational method, Work-related stress, Psychosocial risk factors, Workers’ health

## Abstract

**Background:**

The aim of this work was to introduce a new combined method of subjective and objective measures to assess psychosocial risk factors at work and improve workers’ health and well-being. In the literature most of the research on work-related stress focuses on self-report measures and this work represents the first methodology capable of integrating different sources of data.

**Method:**

An integrated method entitled St.A.R.T. (STress Assessment and Research Toolkit) was used in order to assess psychosocial risk factors and two health outcomes. In particular, a self-report questionnaire combined with an observational structured checklist was administered to 113 workers from an Italian retail company.

**Results:**

The data showed a correlation between subjective data and the rating data of the observational checklist for the psychosocial risk factors related to work contexts such as customer relationship management and customer queue. Conversely, the factors related to work content (workload and boredom) measured with different methods (subjective vs. objective) showed a discrepancy. Furthermore, subjective measures of psychosocial risk factors were more predictive of workers’ psychological health and exhaustion than rating data. The different objective measures played different roles, however, in terms of their influence on the two health outcomes considered.

**Conclusions:**

It is important to integrate self-related assessment of stressors with objective measures for a better understanding of workers’ conditions in the workplace. The method presented could be considered a useful methodology for combining the two measures and differentiating the impact of different psychological risk factors related to work content and context on workers’ health.

## Introduction

The understanding of workers’ stress in the workplace is attracting growing interest in occupational health psychology. Stress, when uncontrolled, has detrimental psychological and physiological effects on workers’ well-being and performance [1]. Increased levels of stress compromise the immune system, reduce cardiovascular functioning, influence blood pressure and hormone excretion and increase the risk of accidents [2]. In this sense, in the Italian context, work-related stress has become a core topic of occupational research, especially after the introduction of the law (D.Lgs 81/08) which obliges organizations to assess psychosocial risk factors and their impact on workers’ well-being in order to prevent strain. Psychosocial risk factors have been defined as aspects related to the planning, organization and management of the job, as well as to the respective environmental and social contexts that have the potential to produce physical, social or psychological damage [3]. These factors can be related to work content and work context.

Workload and repetitive work have been seen as two psychosocial risk factors of work content which are more associated with workers’ strain [4]. Conditions of high workload are related to different negative work outcomes, such as low work satisfaction, burnout and intention to leave the organization [5] and negative health outcomes, such as anxiety, depression and myocardial infarction [6]. Results from recent research have revealed that high workload is also causally related to organizational and behavioural outcomes, like, for example, drug abuse [7], counterproductive work behaviour [8], absenteeism [9], bullying at work [10], low work engagement [11] and reduced job performance [12].

Repetitiveness is a further aspect of the content of work that has a negative influence on strain. Some researchers have shown that repetitive work and a lack of control over the work process constitute a threat to health and well-being and are positively associated with blood pressure [13], physical symptoms and perception of strain [14].

On the other hand, interaction with customers is one psychosocial risk factor of the work context that may be related to dissatisfaction and cause psychological strain [15]. Several researchers underlined that customer management has not been investigated enough as a source of stress in the literature of work-related stress, although 22% of negative events reported by workers occurred when dealing with ‘problem customers’ and some anger-provoking events related to mistreatment by customers [16,17].

In spite of the large body of studies on work-related stress, most of the research has focused on self-report measures to determine both the stressors and the outcome variables [18,19]. Self-reported perceptions may be influenced by workers’ interpretations and this situation has been repeatedly criticized because correlations may be artificially enhanced by conceptual overlap [20] and common method variance [21-23], producing spurious correlations [24].

An alternative methodology is objective assessment based on observational approaches which are independent of workers’ interpretation [25-27]. On the other hand, the same ‘objective’ stressor at work can trigger a serious strain in one person and not in another. As a consequence, the use of objective measures alone cannot be considered as a solution. Starting from these premises, our paper does not aim to test a specific model about stress but the whole discussion is based on the assumption that strain relies on two components, a subjective appraisal and an objective work setting. Main models are compatible with such approach, as Demand-Control (DC) model [28], the Effort – Reward imbalance (ERI model) [29] and the Job-demands resources model [30]. All of them state that, in order to measure the impact of stress, it is necessary to consider workers’ perception.

However, few studies have tried to combine subjective and observational data [31] and compare several kinds of objective assessments with self-reports [32]. One example of the combination of these two measures is the ISTA method (Instrument for Stress-Oriented Task Analysis) [33], which starts from the concept of action regulation, which describes work from a psychological perspective as accomplished by goal-oriented action [34] and tries to match workers’ subjective perception with expert assessment through observation of the workers’ work environment. However, in the ISTA method the subjectivity of the workers was replaced by the subjectivity of the observer, who has to answer to the same self-report rating scales administered to workers observing them. For instance, in order to measure variety (boredom) objectively, the judge evaluates whether the workers receive recurrent and similar tasks (person A) or tasks that are varied and different (person B), using a five-point rating scale ranging from one (‘exactly like A’) to five (‘exactly like B’) [33].

In order to overcome the limitation of the subjectivity of observers, we developed a new observational checklist, in which the variables were operationalized using more observable aspects of content of tasks and context of work. For instance, we have operationalized the variety (boredom) with the observable number of different tasks performed in the 30 minutes of observation, instead of asking the observer to answer on the same rating scale administered to workers with self-report scales.

This observational checklist is one of the tools used by the STress Assessment and Research Toolkit (St.A.R.T.) method which combines the quantitative and qualitative methodological approaches by assessing work-related stressors using different kinds of data: i) organizational archival data (organizational indicators sheet); ii) qualitative data (focus group); iii) worker perception (questionnaire); iv) observational data (observational checklist). The integration of these sources of data can reduce the theoretical and methodological bias related to stress research in the work setting (e.g. common method variance), and allows researchers and professionals to obtain a more reliable description of workers’ stress than through the use of a single analysis tool, providing a more articulate vision of psychosocial risks [35]. Some of these instruments had already validated [36,37].

In this paper, we considered two sources of quantitative data collected using the St.A.R.T. method, that is, observational data collected through a checklist and subjective data collected through an individual self-report questionnaire, and we presented the empirical results of the correspondence and integration of these two kinds of data.

In particular, the aims of this study were:

(1) To examine whether the data collected through the self-report questionnaire were associated with the rating data collected through the observational method in three dimensions: boredom and workload (content of work), and relations with customers (work context);

(2) To verify which psychosocial risk factors, subjective and objective, are related to two workers’ health outcomes: psychological health and emotional exhaustion.

## Method

The research was conducted as a systematic work-related stress assessment of an Italian retail company. The protocol for the research project was approved by the ‘Psychological Research Ethics Committee’ of the Psychology Department of the University of Bologna.

In the first step, the retail shops were sampled by size (small, medium and large) and all employees (total 1,731) of the retail shops chosen actually responded to the interview, although the workers were able to refuse to participate.

After this first step, two organizational positions that were considered key roles in the work organization were chosen for the observation. Subsequently, a subset of 113 workers (about 6.52% of the total number of employees) were identified as targets of direct observation, sampled randomly and stratified on the basis of the size of the retail shops.

The participants were aged between 24 and 59 years old (M = 41.34, sd = 8.03). Their nationality was Italian and 78.8% of the sample was female. With regard to their area of work, 54% of the participants were cashiers and the remainder were catering staff.

The St.A.R.T. (Stress Assessment and Resource Toolkit) method integrated two different instruments in order to assess work-related stress: 1) a structured self-report questionnaire and 2) a structured observational checklist.

### Self-report questionnaire

A self-report questionnaire was administered by interview to 113 workers.

The questionnaire consisted of two parts. The first concerned aspects of the psychosocial work environment, which were assessed with the following validated scales which described factors of the content and context work.

#### *Workload (content of work)*

Workload was measured by the short measure of the Effort of ERI (Effort-Reward Imbalance) scale [38]. Effort consisted of three items referring to the perception of the workload and job pressure (e.g. ‘I have constant time pressure due to a heavy workload’). All items were scored on a four-point Likert scale ranging from one (‘strongly disagree’) to four (‘strongly agree’). Higher scores indicated a greater workload. The Cronbach’s alpha reliability coefficient was 0.51.

#### *Boredom (content of work)*

Boredom was assessed with the adjusted and validated version of Lee’s Job Boredom Scale [39]. The scale contained three items referring to the perception of performing repetitive work activities not commensurate with the worker’s capabilities (e.g. ‘Execute boring and repetitive tasks’). All items were scored on a five-point frequency rating scale ranging from one (‘never’) to five (‘very often’). Higher scores indicated a higher level of perception of repetitiveness. The Cronbach’s alpha reliability coefficient was 0.48.

The scores of reliability for Workload and Boredom were low and below the criterion of .70 [40] (Nunnally & Bernstein, 1994). However, these scales had a low number of items (specifically 3 items) that justified these scores. *Customer relationship management (work context).* Customer relationship management was assessed with a scale created ad hoc. The scale contained three items and referred to the perception of difficulties in relations with customers (e.g. ‘The management of relations with customers is frustrating’, ‘I happen to have discussions with customers’, ‘I feel under stress because of customers’). All items were scored on a five-point frequency rating scale ranging from one (‘never’) to five (‘very often’). An Exploratory Factor Analysis (EFA) through principal component factoring method produced one factor that explained the 56.40% of the variance showing a unidimensional scale. The correlation of the *Customer relationship management* with the self-report measure of workload (.21), as presented in Table 1, is much lower than the correlation with the self-report measure of boredom (.42). This result allowed us to exclude the overlap between the items of the scale of *Customer relationship management* and the items of workload.

**Table 1:** Example of observational checklist

**DIMENSION**	**DESCRIPTION**	**EXAMPLES**	**ASSESSMENT**	**TOTAL NUMBER**
**WORKLOAD**	How many customers are served during the observation? (in the ‘Assessment’ column mark with an X every time a customer is served by observed worker)			N. of customers:
**VARIETY**	In general, how many different operations are performed during the working activity? (in the ‘Assessment’ column mark with an X each operation that you see)	- To pass the fidelity card under the optical reader.		N. of times:
		- To pass the items under the optical reader.		N. of times:
		- To fill a shopping bag.		N. of times:
		- To get cash payment.		N. of times:
		- To get payment by credit card.		N. of times:
		- To count money (coins and banknotes).		N. of times:
		- To get in a supply of coins and banknotes at the checkout.		N. of times:
		- Demagnetize anti-shoplifting device.		N. of times:
		- To put price tag on the item.		N. of times:
		- To receive discount voucher.		N. of times:
		-…………………		N. of times:
		-…………………		N. of times:
**CUSTOMER RELATIONSHIP MANAGEMENT**	Number of customers in a line (in the ‘Assessment’ column mark the number of customers four times in the minute indicated)		T0: …(Time 0)	
			T1: …(after 10 minutes)	
			T2: …(after 20 minutes)	
			T3:…(after 30 minutes)	

Higher scores indicated a higher frequency in problems and difficulties perceived in the relationship with customers. The Cronbach’s alpha reliability coefficient was 0.61.

The second part of the questionnaire comprised the following two measures of strain.

#### *Psychological health*

This outcome was measured by the General Health Questionnaire (GHQ-12) [41]. The scale referred to the perception of feeling under strain (e.g. ‘Could not overcome difficulties’). All items were scored on a four-point Likert scale ranging from zero (‘not at all’) to three (‘much more than usual’). Higher scores indicated worse psychological health. The Cronbach’s alpha reliability coefficient was 0.90.

#### *Emotional exhaustion*

Emotional exhaustion represents the feeling of being emotionally extended and depleted of one’s resources (e.g. ‘Do you feel run down and drained of physical or emotional energy?’) and was measured with the Italian version of the Maslach Burnout Inventory-General Survey [42,43]. The scale contained five items scored on a seven-point scale ranging from zero (‘never’) to six (‘every day’). Higher scores indicated greater exhaustion. The Cronbach’s alpha reliability coefficient was 0.88.

### Structured observational checklist

A new structured observational checklist was developed in order to assess and observe the same psychosocial risk factors measured in the self-report questionnaire. In particular, this new instrument included some dimensions already present in ISTA (Repetitiveness, Workload, Work Problems in terms of interruption and unexpected events, Social Interaction with colleagues and superiors), but tried to overcome the limits of ISTA related to the subjectivity of the observer [33].

As mentioned in the theoretical introduction, boredom was not measured by a subjective assessment of the judges but by the number of different tasks that workers performed in the period of observation. In order to measure workload, we counted the number of customers served during the period of observation and the number of breaks, instead of using a frequency rating scale ranging from one (‘never’) to five (‘very often’) completed by judges. Furthermore, we introduced the new dimension of Customer Management, which was absent in ISTA but is a core aspect of the retail store. Specifically, in order to observe Customer Management, we counted the number of customers in a line four times during the observation.

In a pilot study [36], the new observational checklist was used by two independent judges and the data showed a good within-group interrater reliability between observers.

On the basis of this data, each participant was observed by only one assessor for 30 minutes. In all retail points, the periods of observation were chosen on the basis of the variability of workers’ activities and levels of workload. The aim of the observation was to examine days of maximum workload, days of least work demand and days of medium workload.

In particular, in a previous study we collected data about the days of maximum workload (Saturday afternoons and product promotion days), days of minimum workload (Sunday mornings) and medium workload (late afternoons and midweek evenings) based on customers who visited, on average, the retail stores in the six months prior to the survey. On the basis of these data, we organized the days of observation including these three levels of work demand for each retail point in order to have a representative sampling of workload.

For this work we have considered the following objective variables measured with the observational checklist:

#### Workload (content of work)

The workload was measured by the number of customers served by cashiers and catering staff in the 30 minutes of observation.

#### Boredom (content of work)

Boredom referred to the variety of tasks. The variety of tasks was measured by the number of different tasks performed during the 30 minutes of observation.

#### Customer relationship management (work context)

Customer relationship management was observed by means of the customer queue. In particular, the observer assessed the number of customers in a line four times: at time zero (T0) when the observation started, at T1 10 minutes after the start of the observation, at T3 20 minutes after the start of the observation, and at T4 at the end of the period of observation. The mean score of customers in a line for the four times was calculated for each worker observed.

### Statistical analysis

The statistical package SPSS (version 18.0) was used to analyze the internal consistency of the scales through Cronbach’s alpha reliability coefficient and the correlations among variables through Pearson’s coefficient.

In order to test which subjective and objective psychosocial risk factors were related to workers’ health outcomes, a hierarchical regression analysis was performed as implemented by SPSS. In the first step of the hierarchical regression, control variables were entered (occupational type, gender and age), followed by the self-report psychosocial risk factors in order to examine their impact on the perception of workers’ health measured by self-report instrument (second step). In the third step of the hierarchical regression we entered the objective measures of the same psychosocial risk factors with the second step.

## Results

Means, standard deviations and intercorrelations among measures are presented in Table 2.

**Table 2:** Descriptive statistics and inter-correlations among all variables

		**Range**	**M**	**D. Std.**	**1**	**2**	**3**	**4**	**5**	**6**	**7**	**8**
Self-Report measures	1. Workload	1-4	2.59	.75	1							
	2. Boredom	1-5	2.71	.90	.17	1						
	3. Customer relationship management	1-5	2.41	.94	.21*	.42**	1					
	4. Psychological health	0-3	0.92	.55	.06	.46**	.50**	1				
	5. Emotional exhaustion	0-6	2.80	1.63	.35**	.56**	.66**	.60**	1			
Objective measures	6. Workload (number of customers served)	0-37	12.42	7.62	-.02	.06	.15	-.03	.13	1		
	7. Boredom (variety of tasks)	2-14	5.91	2.49	.09	-.07	-.18	-.11	-.25**	.37**	1	
	8. Customer relationship management (customer queue)	0-6	1.70	1.29	.00	.02	.27**	.26**	.25**	.41**	-.15	1

*p ≤ 0.01**;** **p ≤ 0.001.

As shown in Table 1, the self-report measure of customer relationship management was correlated with the corresponding objective measure of customer queue. On the other hand, the number of customers served and the variety of tasks, which are aspects of work content, were not related to the corresponding self-report measures of workload and boredom.

Furthermore, some intercorrelations were found within the measures of the same typology. For example, within the subjective measures, customer relationship management was associated with both boredom and workload and, within the objective measures, the number of customers served had significant relations with the other two objective variables.

In order to examine which dimensions (self-report and objective) were related to the health outcomes psychological health and emotional exhaustion, two hierarchical regression analyses were performed. Findings are presented in Table 3 in relation to each of the two health outcomes considered.

**Table 3:** Hierarchical regressions

	**Psychological health**			**Emotional exhaustion**		
	**Step1**	**Step2**	**Step3**	**Step 1**	**Step2**	**Step3**
Predictors	*β*	*β*	*β*		*β*	*β*
**Control variables**						
Gender	.10	.04	.07	.09	.03	.07
Age	.04	.07	.03	.04	.05	.05
Occupational type	-.12	-.04	-.14	-.17	-.08	-.05
**Self-report measures**						
Workload		-.07	-.06		.18**	.21**
Boredom		.31***	.34***		.34***	.35***
Customer relationship management		.39***	.33***		.48***	.41***
**Objective measures**						
Workload (Number of customers served)			-.27*			-.07
Boredom (Variety of tasks)			-.08			-.18*
Customer relationship management (Customer queue)			.27*			.13
R^2^ adjusted	.006	.31	.38	.02	.56	.59
ΔR^2^		.25**	.07**		.54**	.03^*^

*p ≤ 0.05**;** **p ≤ 0.01; ***p ≤ 0.001.

As shown in Table 2, both self-report and observational measures were predictive of psychological health. In fact, self-report measures explained 34% of the variance of psychological health and the increase of variance explained by the observational measures was significant (*ΔR*^*2*^ = 0.07; *Δ F*(3,113) = 5.01; p ≤ 0.01).

Their impact was different, however, depending on the three dimensions considered. In particular, the dimension of customer relationship (related to the work context) influenced psychological health in both measures (self vs. observational) whereas only the self-report measure of boredom and the observational measure of workload were predictive of workers’ health.

Regarding the second health outcome considered, both subjective and observational measures were predictive of emotional exhaustion, explaining 61% of the variance. In particular, subjective measures explained 57% of the variance and the increase in variance explained by the observational measures was significant, although it was lower than that of psychological health (*ΔR*^*2*^ = 0.04; *Δ F*(3,113) = 3.11; p ≤ 0.05). If we examine the three dimensions considered in each of the two steps, the beta coefficients of all the self-report measures were significant whereas only the objective measure of boredom (variety of task) was significantly associated with emotional exhaustion.

## Discussion

The literature on occupational stress underlines that it is important to rely on different sources of data collection in order to evaluate work-related stress properly [44]. In this sense, the St.A.R.T. method represents a multi-source approach that, on the one hand, may overcome the dichotomy between subjective (self-report questionnaire) and objective (observational checklist) measures and, on the other hand, proposes mixing quantitative (i.e. archival data, observational data) and qualitative (self-report questionnaires and focus groups) methods in order to compensate for their mutual weaknesses. In this study we focused on the first dichotomy, considering the relationship between two different types of quantitative data: self-report data collected through an individual questionnaire and rating data collected through an observational checklist. In fact, the pivotal aim of our work was to reduce methodological bias, related to separate assessment of objective (i.e. observational) and subjective (questionnaire) data. First of all, the findings of this work showed that the two measures (self-report vs. observational) of the dimensions of job content (workload and boredom) were not interrelated, whereas the psychological factor related to job context (customer relationship management) showed a correlation between the measures of the two different tools.

This means that objective aspects related to the work tasks, such as an elevated level of repetitiveness and work demands in terms of numbers of customers that workers have to serve, are not sufficient to determine a perception of high boredom and high workload in the workers. For this reason it is important to use both the measures because some psychological risk factors are not demanding of themselves and do not automatically lead to the process of stress. The perception of high demand depends on personal and resource characteristics and the attributional process activated between stressors and individual response [45]. This absence of interrelation between subjective and objective data in the content of work will be well interpreted in the future using the qualitative data in the triangulation design [46] forecast by the St.A.R.T. method.

As presented above, however, the dimension of work context related to customer relationship management showed correlations between the subjective and objective measures. It is apparent that the objective measure of customer queue, often related to the inadequate management of the retail store and the absence of organizational support, is sufficient to cause the difficult relationship with customers and the consequent emotional effort that these workers perceived. Furthermore, these two measures predicted the psychological health perceived by workers in this study. This result could have an important applicative outcome because the customer queue is easy for retail stores to control after accurate study of workload curves in order to prevent their workers experiencing strain.

Another interesting finding concerned the impact of subjective and objective dimensions on the two health outcomes considered. The data showed that the self-report measures were associated more with psychological health and emotional exhaustion than objective data, although the amount of variance increased significantly when objective measures were entered in the hierarchical regression.

This result is understandable considering that people use different coping strategies to face work demands and potential objective stressors, and obtain different outcomes for the perceptions of tasks and, consequently, for health.

Finally, within the objective measures, the three dimensions predicted psychological health and emotional exhaustion in different ways. The number of clients served and the customer queue were associated with psychological health whereas the variety of tasks predicted emotional exhaustion.

In this context, the introduction of an intervention in the organization of work activities, such as job enlargement, could prevent the feelings of being emotionally extended and depleted of personal resources.

The present study is limited by several factors. First of all, the participants belonged to a specific company and it would be necessary to extend the study to other companies in order to corroborate the validity of the instrument. Second, the St.A.R.T. method is one of the first to compare self-report data with observational objective data and future research will be necessary to analyse the correspondence between different sources of data. Third, we measured only some psychological risk factors and it might be useful to introduce new elements of work content and context that are related to the psychological health and emotional exhaustion of workers.

## Conclusion

The only use of self report measures represents one of the main limitations of stress evaluation. For this reason we think that it is extremely important that the St.A.R.T method uses and verifies two typologies of measures. The St.A.R.T. method presented in this work appears to be able to reduce theoretical and methodological bias typical of stress research in work settings, and allows researchers and professionals to obtain a more reliable description of work-related stress than with the use of a single analytical tool. In particular, this methodology allows researchers to compare self-report and objective data of the same dimensions of work content and context and is able to differentiate their effect on health outcomes.

The results of this study suggest the use of an integrated approach as the best method to measure occupational stress, providing a more articulated vision of psychosocial risks. In fact, there is evidence to support the theory that objective measurement alone is not enough to assess work-related stress and to manage the assessment process from a prevention perspective. On the other hand, studying psychosocial risk factors by means of subjective tools only is likely to produce measurement bias resulting from the subjectivity of personal interpretations of risk factors.

In summary, the use of the St.A.R.T. method represents a first attempt at assessing work-related stress, overcoming the limitations of most research on psychosocial risk factors that is based on the use of a single measure.

## Authors’ contribution

CP was involved in the conception and design, acquisition, analysis and interpretation of data and drafting of the manuscript. DG was involved in the conception and design, analysis and interpretation of data, drafting of the manuscript and supervision. AR was involved in the acquisition, analysis and interpretation of data and drafting of the manuscript. MCT was involved in the conception and design, acquisition and interpretation of the data. FSV was involved in the conception and design, interpretation of the data and supervision. All authors read and approved the final manuscript.
